# Low-Cost Human–Machine Interface for Computer Control with Facial Landmark Detection and Voice Commands

**DOI:** 10.3390/s22239279

**Published:** 2022-11-29

**Authors:** Pablo Ramos, Mireya Zapata, Kevin Valencia, Vanessa Vargas, Carlos Ramos-Galarza

**Affiliations:** 1 Departamento de Eléctrica, Electrónica y Telecomunicaciones, Universidad de las Fuerzas Armadas ESPE, Av. General Rumiñahui S/N y Ambato, Sangolquí 171103, Ecuador; 2 Centro de Investigación en Mecatrónica y Sistemas Interactivos-MIST, Universidad Indoamérica, Av. Machala y Sabanilla, Quito 170103, Ecuador; 3 Facultad de Psicología, Pontificia Universidad Católica del Ecuador, Av. 12 de Octubre y Roca, Quito 170143, Ecuador

**Keywords:** H/M interface, mouse, keyboard, face tracking, facial landmarks, speech recognition, voice commands, handicap

## Abstract

Nowadays, daily life involves the extensive use of computers, since human beings are immersed in a technological society. Therefore, it is mandatory to interact with computers, which represents a true disadvantage for people with upper limb disabilities. In this context, this work aims to develop an interface for emulating mouse and keyboard functions (EMKEY) by applying concepts of artificial vision and voice recognition to replace the use of hands. Pointer control is achieved by head movement, whereas voice recognition is used to perform interface functionalities, including speech-to-text transcription. To evaluate the interface’s usability and usefulness, two studies were carried out. The first study was performed with 30 participants without physical disabilities. Throughout this study, there were significant correlations found between the emulator’s usability and aspects such as adaptability, execution time, and the participant’s age. In the second study, the use of the emulator was analyzed by four participants with motor disabilities. It was found that the interface was best used by the participant with cerebral palsy, followed by the participants with upper limb paralysis, spina bifida, and muscular dystrophy. In general, the results show that the proposed interface is easy to use, practical, fairly accurate, and works on a wide range of computers.

## 1. Introduction

Significant technological advances in the field of artificial intelligence have boosted the use of computers everywhere. Moreover, a large increase in computing capacity allows us to carry out tasks in an unimaginably short time. Therefore, interaction with computers is becoming essential for daily activities such as working, studying, distance learning, running a business, gaming, etc. For the disabled who cannot operate a computer, this fact reduces their integration into society. Consequently, it is important to minimize this effect by developing and implementing techniques that allow disabled people to overcome this barrier.

One of the main objectives is to help people with motor disabilities use computers, since most peripheral computing devices are controlled by the body’s upper extremities. By taking advantage of modern computers’ sophisticated features, it is possible to find a solution. For instance, computer vision is one of the fields that have benefited the most from computer improvements. This feature has been used in a variety of applications that require a computer to emulate the eyesight of a human in order to observe, analyze, and control the environment. These applications include activities involved in a social environment [1], where the computer is expected to perform tasks that would be complicated for a human being, potentially making people’s lives easier.

Considering the successful applications of computer vision in other fields, various techniques derived from it have been developed to deal with the problem under study. Refs. [2,3,4,5,6] apply the concept of eye tracking to facilitate computer control by means of gaze detection. However, the disadvantage of this technique is the use of external devices, such as glasses, which can be obtrusive and costly for users. Other works propose systems based on face tracking [7,8,9,10], in which computers can be controlled with gestures and head movements. Nevertheless, their use is limited to specific applications.

On the whole, the use of computers has become essential, not only for interacting with the world through the global internet network, but also for accomplishing educational and work activities. For this reason, it is imperative to develop a low-cost solution that can be integrated into a generic computer. Therefore, this work proposes an interface called EMKEY, which controls mouse actions by face and voice recognition.

The interface is intended to work on a wide range of computers. Pointer control is achieved by means of head movement, using the nose as the reference point. Different voice commands are used to emulate mouse actions and speed up pointer movement by screen segmentation. Additionally, a dictation mode is available, without the need for an internet connection. This feature allows the conversion of speech to text, fulfilling some keyboard functions.

The main contribution of this work is providing people with motor disabilities with a low-cost, versatile, and fairly accurate mouse and keyboard emulator, adaptable to standard computers, to facilitate their access to the internet and computer applications. In order to validate this proposal, two types of tests were conducted: (a) performance tests, carried out under different environmental conditions and with various computer features; and (b) user tests, to demonstrate the usability and usefulness of the proposal.

The remainder of the article is organized as follows. Section 2 presents related work. Section 3 details the interface design. Section 4 describes the materials and method. In Section 5, the different tests carried out in this study and their results are presented. Section 6 presents a discussion comparing the obtained results with those of similar works. Finally, Section 7 concludes this article.

## 2. Related Work

This section summarizes some relevant works dealing with techniques to diminish the barriers faced by people with upper limbs disabilities when using computers.

Lund et al. [11] designed a prototype of a system to control the mouse and keyboard through an encapsulated intraoral device that associates the movement of the cursor to that of the tongue. A more recent work [12] presented a system similar to the previous one, where the tongue is used to emulate mouse movements. It consists of a multimodal interface mounted on a device similar to a hearing aid, which allows people with disabilities to use the computer. Using the tongue, the user can perform the clicking action. The movement of the cursor is proportional to that of the user’s head, and via voice recognition technology, typing on the keyboard can be emulated. In another work [13], a way to emulate the operation of the mouse by means of movement and pressure sensors for performing actions such as pointer movement and clicks is presented.

McFarland et al. [14] proposed a system that captures brainwave patterns to perform different actions with the cursor, such as moving it up, down, right and left. Additionally, it enables options to select, drag, and drop items. In other research [15], a human–machine interface was developed using a device called Emotiv EPOC+, which allows the computer to be controlled through facial expressions and movement sensors. This device is a brain–computer interface that acquires and processes electroencephalography (EEC) signals from the brain and from the muscles of the face using electromyography (EMG). The movement of the face is proportional to that of the cursor, and to emulate the functionalities of the mouse, different facial expressions are used.

Lupu et al., developed a system to control the computer pointer by using eye tracking and external devices, such as a webcam and video glasses with infrared LEDs [4]. The two components are coupled to allow gaze detection at a distance of approximately ten centimeters. Another proposal based on eye tracking has been presented [5], in which a software that works in parallel with an eye-tracker device was developed. It uses the “mouse cursor control” function of the device itself. Furthermore, with the detection of the gaze on a fixed point for a specific time, certain actions, such as clicking, can be performed. The system also incorporates a virtual keyboard that allows users to enter text depending on gaze position. Espada et al. implemented an eye-scanning system for a girl with severe cerebral palsy in Spain. This work aimed to make the learning process more effective and inclusive by improving her social and communication skills [3]. In this case study, positive results were evidenced. In a work by Sias et al., a low-cost system that allows emulating mouse operation by means of head movement and gaze detection is presented [6]. In this work, glasses that incorporate a gyroscope and an accelerometer were used. The pointer moves along the screen, controlled by the implemented sensors. The click action is performed by eye-tracking algorithms, verifying whether the gaze dwells on a single point for a certain time.

Loewenich et al. [16] combined facial detection with voice recognition to enable the control of a computer by means of head movements, although with a limited list of voice commands. A similar proposal was developed [17] through a system that consists of two modules. The first is the software module, which is in charge of continuously comparing previous features of the face with new ones. If the system detects any movement, it is translated into pointer movement. The second module employs voice commands, whereby it is possible to emulate mouse actions such as click, double-click, right-click, etc. Additionally, specific commands are implemented to open specific applications such as Word, Paint, and Excel, among others. In Guiawal et al. [18], the mouse pointer is controlled by following the nose and performs actions such as clicking or double-clicking based on the analysis of facial gestures. Khan et al. [19] also use this technique to achieve the movement of the mouse, but the click function is performed by detection of the teeth. Gupta et al. [20] present a system to control some common computer actions through head movements and voice commands. In this proposal, users had to pre-record twelve voice commands in the Bangla language so that the functions associated with each command could be executed.

Several studies [8,9,10] have proposed the use of face tracking for specific applications such as Facebook, Google Chrome, or WhatsApp Web. The mouse functionalities are controlled using facial gestures and head movements. In one such work [9], a complete interface was developed, adding voice commands to facilitate the execution of some functionalities in the Google Chrome application, such as surfing the internet or checking email. In addition, the system has a dictation mode that facilitates transcription to text, which is useful for sending messages or making queries. Elsewhere [21], a face-tracking system based on convolutional neural networks was proposed to control a computer mouse. Two neural networks were trained. The first uses images of faces making horizontal and vertical movements to achieve cursor movement; the second uses images of faces with opened and closed eyes to control click action.

Darabkh et al. [22] developed a system that works exclusively with voice recognition. Initially, a dataset containing the characteristics of the audio signal corresponding to each voice command must be configured. Through these commands, it is possible to perform actions that include opening specific applications, recording documents, and emulating mouse functionalities, among others. Alhamzawi et al. [23] proposed a system to control the computer cursor through computer vision algorithms. A blue dot that is painted on a sports band placed on the forehead is detected. The movement of the head is proportional to that of the cursor. In addition, to achieve a click, the user must keep the head fixed for a time *n*; to perform a double click, the time is *n* + 1. In another specific approach [24], speech recognition and gaze tracking are used to generate a system focused on programmers with disabilities. The interface is used mainly via voice commands to perform actions that facilitate navigation in a development environment. By means of a virtual keyboard and gaze detection, code can be typed. Lastly, some works in the literature [25,26] aim to provide a way for people with disabilities to interact with devices other than computers, such as cell phones or tablets.

Considering that the use of techniques requiring external devices such as glasses, sensors, or electrodes are expensive and obtrusive, this paper presents an application interface based on face tracking and voice recognition that emulates mouse and keyboard functionalities. The proposal is a low-cost solution that could be implemented on a generic computer.

## 3. EMKEY Application

**Figure 1:**
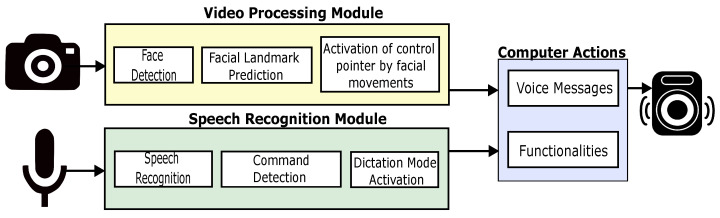
EMKEY block diagram.

The EMKEY application is proposed as a generic interface to facilitate the use of a computer by people with motor disabilities. This interface enables the control of mouse actions, emulating some common keyboard shortcuts, transcribing speech to text, and alerting users of some specific actions. Figure 1 describes the design and implementation of EMKEY.

This interface is composed of three modules. Two of them are responsible for acquiring and processing the video and speech signals that will be used to control the application, as shown in Figure 1. The first one is the video processing module, which is dedicated to recognizing facial movements to control pointer positioning. The second module is devoted to voice command recognition and transcription to text. Depending on the recognized command, the interface performs a predefined task related to a mouse action, shortcut key, or application functionality. In addition, this module implements a function that performs the transcription of dictation. The last module plays pre-recorded messages alerting the user of a specific event.

**Figure 2:**
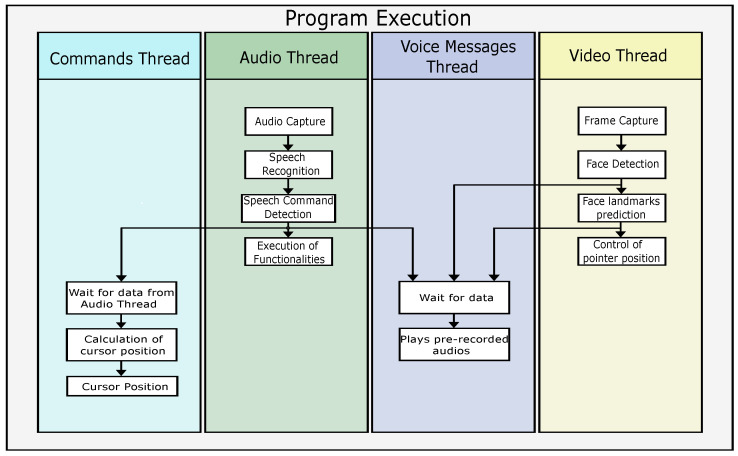
Program execution scheme.

The application was designed to be implemented using multi-threading, where each module has one thread. In addition, the speech-to-text module has an auxiliary thread called “Command Thread” that calculates the cursor’s position. In Figure 2, the execution scheme of the program is presented, specifying the threads that are executed simultaneously and the data transmission between them. The functions performed by each thread are also specified. In the video thread, the user’s image is captured continuously, while the audio thread allows the system to listen continuously to detect commands.

It is important to state that the EMKEY application was designed to be used by Spanish speakers. However, it could be easily adapted to other languages. The proposed interface was developed entirely using the Python programming language to take advantage of the large number of libraries and modules provided by the Python community. The following sections explains each module in detail.

### 3.1. Video Processing Module

**Figure 3:**
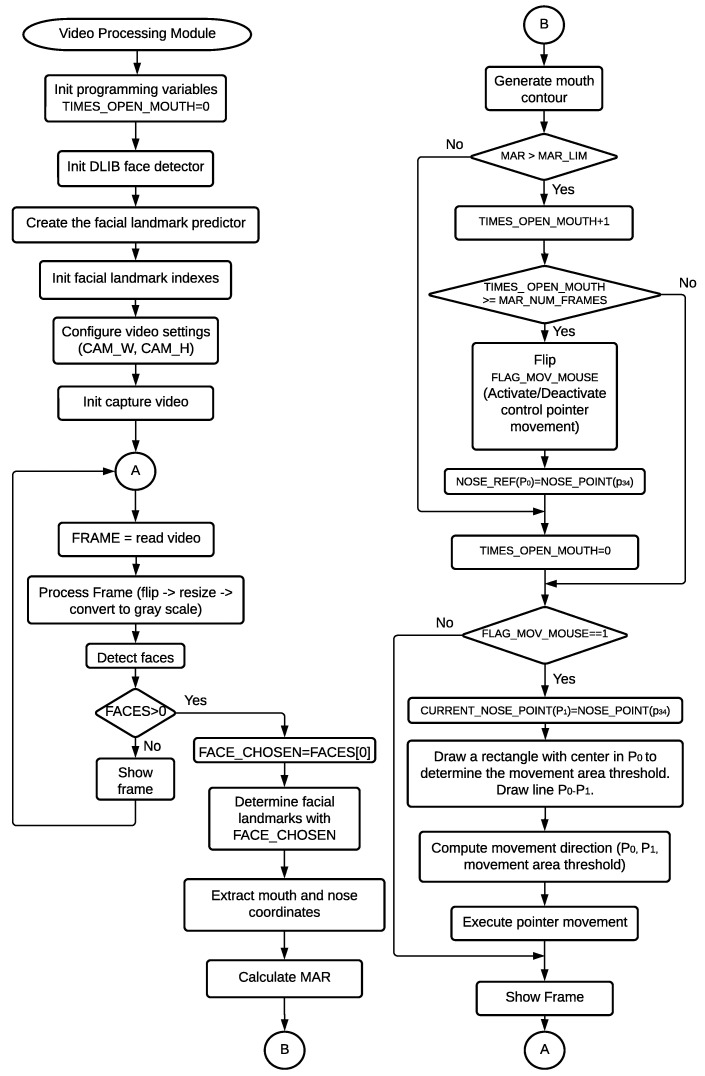
General flowchart of the video processing module. Note: The programming constants are: *CAM_W* = 640, *CAM_H* = 480, MAR_LIM = 0.6, MAR_NUM_FRAMES = 15.

The video processing module executes three main actions: face detection, facial landmark recognition, and control of pointer position. The proposed face detector is based on the use of the Dlib library [27,28] and complemented by a predictor of facial landmarks [29], implemented in the same library. It provides 68 coordinates that describe different areas of the face, such as the mouth, nose, eyes, and contour. If an open mouth is detected, the module enables or disables the control of the cursor position by facial movements. It should be noted that both models provided by the Dlib library—the facial recognition model and the facial landmark predictor—are already trained. Thus, no training has been performed in this work. Figure 3 shows a general flowchart describing how the program executes when each frame is analyzed. It should be noted that this module interacts in parallel with the speech recognition module; both end their execution when the user pronounces the “turn off” command.

**Figure 4:**
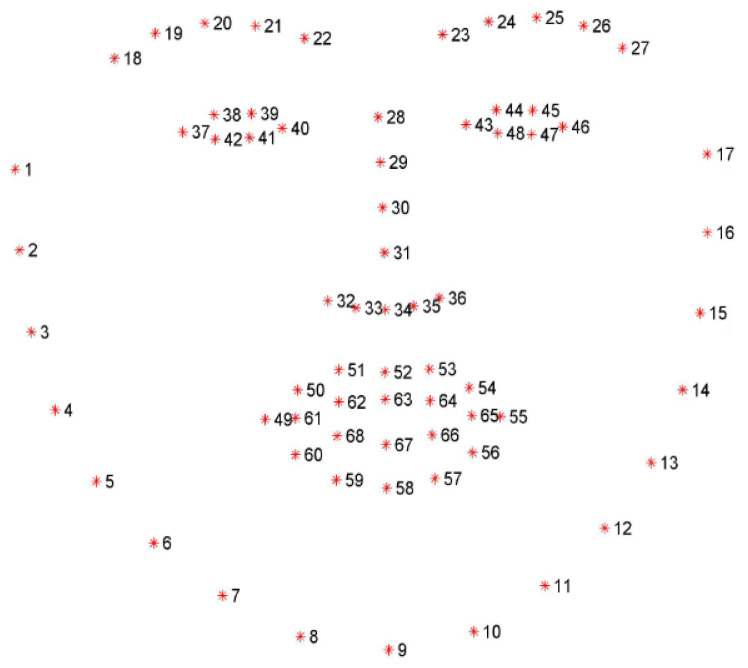
Facial landmarks acquired from Dlib predictor. Each landmark point determine a specific part of the face [29].

The procedure initiates with the reception of images captured by the camera. The images then pass through a pre-processing stage where they are transformed into grayscale. Additionally, a horizontal reflection is made. If two or more faces are detected, the video thread sends an event to the voice alert thread to activate a message that indicates that more than one face has been detected. Next, the facial landmark prediction searches for the coordinates corresponding to the mouth and the center of the nose of the first face detected (point 34) (Figure 4). If an open mouth is detected for 15 consecutive analysis frames, control of the cursor position is either enabled or disabled.

In order to determine if the mouth is open, a relationship between the coordinates of the points $p_{61}$ to $p_{68}$ of the facial landmarks is assessed. These points correspond to the inner section of the mouth. First of all, the distance between parallel coordinates of the mouth is calculated. Coordinates $p_{62}$ and $p_{68}$, $p_{63}$ and $p_{67}$, $p_{64}$ and $p_{66}$ are used to determine the height of the mouth; the distance between $p_{61}$ and $p_{65}$ defines the width of the mouth. Secondly, the Mouth Aspect Ratio (MAR) presented in Equation 1 is calculated. This equation is based on the concept known as the Eye Aspect Ratio (EAR) [30]. The MAR value will vary depending on the position of the lips. When the user opens his mouth, a threshold value established at 0.6 will be exceeded, indicating an open mouth, where $p_{61}$–$p_{68}$ are the coordinates shown in Figure 4, corresponding to the lower part of the lips.

(1) $$MAR=\frac{∥p_{62}-p_{68}∥+∥p_{63}-p_{67}∥+∥p_{64}-p_{66}∥}{2∥p_{61}-p_{65}∥}$$

**Figure 5:**
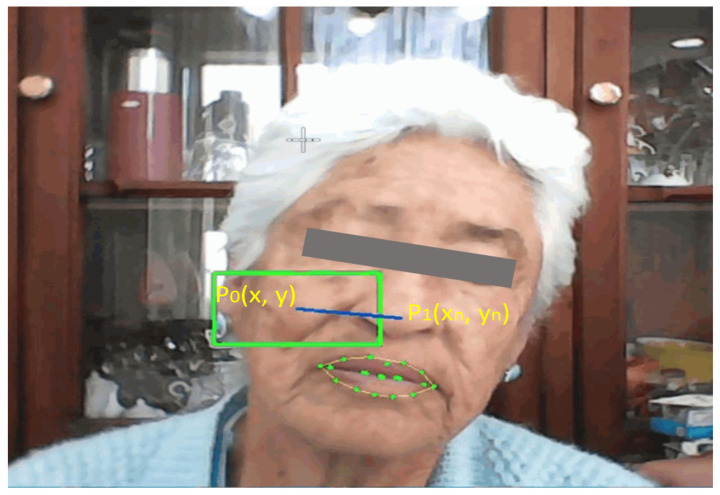
Face recognition when moving to the left.

If the user maintains an open mouth for more than the time it takes to capture 15 consecutive frames, the activation state of the control of pointer position is flipped. Immediately, an event notification is sent to the voice alert module to play the corresponding message. If the pointer control is activated, the position of the central coordinate of the nose ($p_{34}$) is captured and saved in $P_{0}\left(x,y\right)$. It will be used as a reference point for later calculations to determine cursor movements. Pointer movement is achieved by applying a function in which $P_{1}$($x_{n}$, $y_{n}$) is compared with the previously defined reference point $P_{0}$. In Figure 5, it can be seen that a green rectangle with dimensions of 60 × 30 and a segment $P_{0}-P_{1}$ (blue line) are drawn on the user’s face to generate a reference for the pointer movement. When $P_{1}$ surpasses the limits of the rectangle, the pointer will move in the direction from which it came (up, down, right, or left).

The pointer speed (5 pixels/frame) was set to make it easier to select small objects on the screen, so it may seem slow at first glance. To compensate for this disadvantage, voice commands are used to directly position the pointer on twelve different segments of the screen. In addition, to avoid failures generated by sudden head movements, it is possible to reset the pointer position by opening the mouth again.

### 3.2. Speech Recognition Module

This module is in charge of tasks related to voice recognition, command detection, and dictation/transcription. This block receives audio signals captured through a microphone and transforms them into text. The generated strings are compared to words or phrases in order to launch the activation of an interface command. To work with voice recognition, the *Vosk* library [31] was used. It does not need an internet connection since it works with two pre-trained models: (1) a language model, which describes sequences of words in the same language; and (2) an acoustic model, which represents the sounds of the language. In this case, it was necessary to download pre-trained models that describe the audible signals and the coherence between words in the Spanish language.

**Figure 6:**
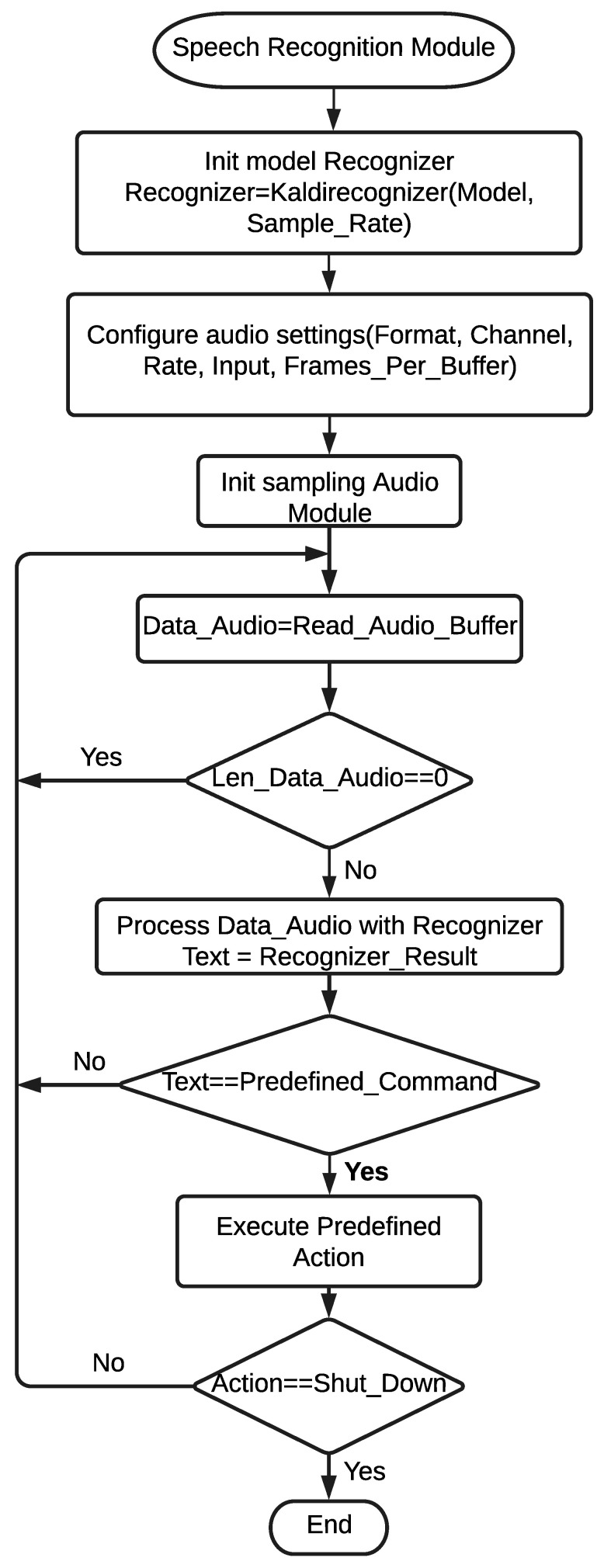
General flowchart of speech recognition module. Note: the parameter values are MODEL = Spanish, Sample Rate = 16 k, Format = 16 Bits, Channel = 1, Rate = 16 k, Input = True, and Frames_Per_Buffer = 8192.

Voice commands are used to set the pointer position, emulate mouse functionalities, control windows, close the application, and transcribe dictations. If what the user says coincides with any of the commands, the respective function will be executed. If the dictation/transcription mode has been activated, the user will also be able to use the commands shown in Table 1. In addition, when the user enables/disables the transcription mode, a notification is sent to the alert message module to play the corresponding message. Figure 6 shows graphically the general execution flow of the speech recognition module.

#### Command Execution

**Figure 7:**
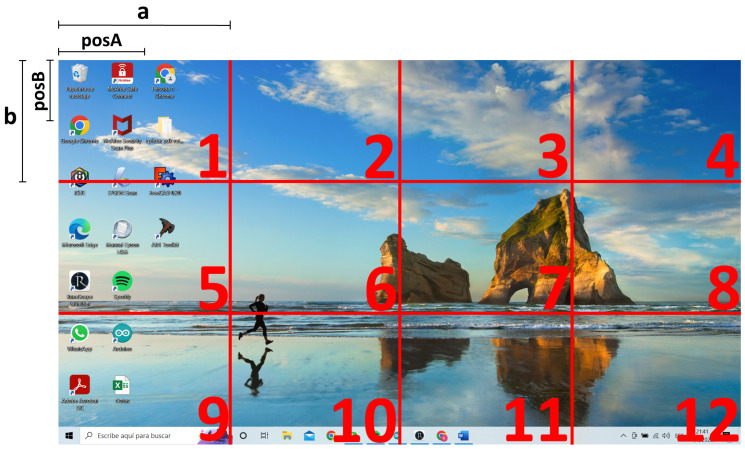
Virtual segmentation of the screen to facilitate control pointer position (quadrant number). “a” is a quarter of the screen width, “b” is a third of the screen height, “posA” is half of “a”, and “posB” is half of “b”.

Since most of the interface functionalities are related to the mouse and keyboard functions, the *Pyautogui* library [32] was used. To facilitate the control of the pointer position, the screen was segmented into twelve quadrants, as shown in Figure 7. When the user says a number from one to twelve, the pointer will automatically move to the center of the designated area. The list of custom control voice commands is shown in Table 2.

To calculate the exact coordinates of the quadrant of the screen, the dimensions of the screen are extracted and a specific formula is applied depending on the quadrant row where the user wants to position the pointer. Figure 7 illustrates the parameters required to calculate the coordinates. “a” is a quarter of the screen width, “b” is a third of the screen height, “posA” is half of “a”, and “posB” is half of “b”. These values depend on the screen resolution.

- If the user says a number from one to four, the coordinates are calculated by Equation (2):
  (2) {posA+[a(#quadrant−1)],posB}
- The calculated coordinates for commands from five to eight are obtained by Equation (3):
  (3) {posA+[a(#quadrant−5)],posB+b}
- Commands from nine to twelve use Equation (4) to calculate the coordinates:
  (4) {posA+[a(#quadrant−9)],posB+2b}

As stated above, when the dictation/transcription mode is activated, other specific commands can be used. These are summarized in Table 1.

### 3.3. Voice Alert Module

The voice alert module is used to warn the user about a particular event. This module receives signals from the video processing module or the speech recognition module to play a pre-recorded MP3 file by using the *Pygame* library [33]. As mentioned above, four situations activate this module:

- When multiple faces are detected: the user will hear “caution, more than one face detected”;
- When the user’s mouth is opened: if the pointer movement is disabled, the user will hear “mouse on”, otherwise “mouse off”;
- When the user enables the dictation mode: the user will hear “dictation on”;
- When the user disables the dictation mode: the user will hear “dictation off”.

**Figure 8:**
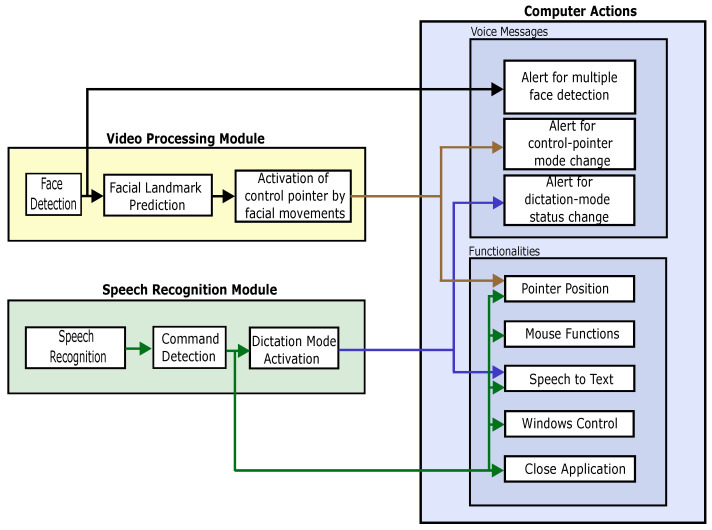
EMKEY module interactions.

To conclude this system description, Figure 8 summarizes the interaction between the different modules of the EMKEY application.

## 4. Materials and Method

This section describes the development platform used to implement the EMKEY emulator as well as the methodology used to evaluate its functionality and usability. The evaluation consists of two types of tests. The first, which is an experimental test, aims to assess the emulator under different environmental and technical conditions in order to determine the minimum requirements for it to run effectively. The second test consists of non-experimental quantitative research that uses instrument-based questions for data collection. The survey is based on eight close-ended questions intended to determine the usefulness and usability of the emulator. The survey was addressed to two groups of people: with and without disabilities. Data analysis was conducted using a correlational design to describe and measure the relationship between variables found in the questions of the survey.

### 4.1. Development Platform

In order to develop the interface, a laptop equipped with an Intel Core I5-8250U processor and 8 GB RAM was used. It also had Intel UHD graphics and an integrated standard VGA webcam. For capturing audio, an external Genius MIC-01A microphone with a frequency range between 100 and 11,000 Hz was employed.

To develop the application, *Python* was selected as the programming language, since it is free. Python also has the ability to generate executable applications oriented to different operating systems, such as Windows, Mac, or Linux. The main Python libraries used are the following:

- *OpenCV*: to capture video and perform the prepossessing stage;
- *Dlib*: to access pre-trained models to detect faces and predict landmarks;
- *Vosk*: to recognize voices and generate the speech to-text-function;
- *Pyautogui*: to access the mouse and keyboard functionalities;
- *Pygame*: to play the MP3 audios.

Once the EMKEY emulator passed the functional tests, it was be ready to be evaluated by users.

### 4.2. Testing Methodology

As previously mentioned, the EMKEY application was tested from two points of view. Firstly, the emulator performance was evaluated considering different environmental and technical features detailed as follows:

(a) Different environmental lighting conditions;
(b) More than one face detected by the camera;
(c) Different levels of environmental noise;
(d) Different hardware and software features of the computer.

Second, the emulator’s usability and usefulness were evaluated. To achieve this, a survey was designed to gather feedback on each user’s experience after using the emulator. In order to avoid bias in the results, people of different ages, genders, and abilities were selected. Additionally, two studies with different target subjects were conducted:

(a) Study 1: people of different ages and genders without disability;
(b) Study 2: people with different degrees of motor disability.

In Study 1, the sample comprised 30 participants. The results of the test provide information about the usability of the emulator. In Study 2, the sample comprised four participants due to the difficulty of recruiting. Despite the size of the sample, the results provide good feedback on the usability and usefulness of the emulator.

Table 3 lists the questions of the survey. Questions Q1 to Q6 are evaluated on a numeric rating scale from 1 ({Not Satisfied) to 5 (Very Satisfied), whereas Q7 and Q8 collect numerical data. 

For the group with different degrees of motor disability, the last two questions of the survey were slightly changed to better adapt them to their situation, as shown in Table 4.

In Study 1, to analyze if the usability of the emulator depends on age or gender, five hypotheses were proposed. Each one was associated with two variables.

**Hypothesis 1.**  *The level of previous computer skills is associated with adaptation to the emulator.*

**Hypothesis 2.**  *The time it takes to perform an internet search is associated with the age of the participants.*

**Hypothesis 3.**  *The participant’s age is related to the number of times that the pointer movement must be restarted by holding the mouth open to perform the exercise.*

**Hypothesis 4.**  *Age is associated with the degree of ease using the emulator.*

**Hypothesis 5.**  *The performance of men and women using the emulator is similar.*

Through this exploratory study, the correlation (*r*) between different variables involved in the survey was determined to validate the hypotheses. Regarding Study 2, the usability of the emulator based on the opinion of people with disabilities was computed by summing up the score for responses Q1 to Q6 and Q8b. This result, which ranges up to 35 points, also provides feedback on the usefulness of the emulator for people with motor disabilities.

### 4.3. Experimental Protocol

To run the empirical test, each participant was asked to follow the protocol below:

- Participate in an induction procedure.;
- Familiarize yourself with the interface;
- Execute a predefined task;
- Fill out a survey.

First, the participant received induction training on how EMKEY works and how to control its functionalities through face movements and voice commands. Then, the participant was given a user manual that includes the list of the voice commands and a printed card illustrating the screen segmentation to position the pointer using voice commands. Next, the participant was asked to interact with the computer via EMKEY long enough to become familiar with its functionalities. After that, the participant was requested to open a web browser, make the following query: “World’s most populated country”, and open the first link that appears. This task aimed to test the main application functionalities related to the control pointer and dictation/transcription mode. In the end, the participants answered an eight-question survey (see Table 3) related to aspects such as difficulty, functionality, and adaptation. As stated above, two questions were modified for participants with disabilities (see Table 4).

## 5. Results

### 5.1. System Testing under Different Scenarios

This section presents the functional test of the emulator under different environmental and technical features. Performance tests were carried out under different lighting levels and with multiple faces in the captured frame. Finally, tests were conducted on computers with different hardware and operating systems.

#### 5.1.1. Lighting Level Test

**Figure 9:**
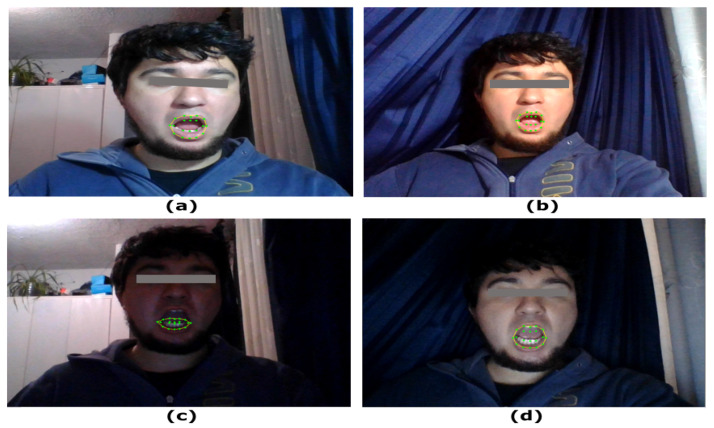
Face detection and mouth landmark prediction at different lighting levels. (**a**) Image captured at 59 lux. (**b**) Image captured at 32 lux. (**c**) Image captured at 24 lux. (**d**) Image captured at 2 lux.

The interface was tested with four different lighting levels, varying from 59 to 2 lux. In order to ensure adequate operation of the video module, the prediction of mouth coordinates should be as accurate as possible. In Figure 9, cases a, b, and d provide an accurate prediction of the mouth coordinates. On the other hand, case c give an erroneous prediction. Comparing the four images, it can be seen that in cases where the face is brighter, the prediction of facial landmarks improves considerably. However, in dark environments with a clear image of the face, as in (d), the system is able to work properly. From this analysis, it was determined that the lighting level of the environment is less relevant than the lighting and focus of the face.

#### 5.1.2. Multiple Faces in a Frame

This test evaluates the behavior of the emulator when multiple faces are detected. The face detection algorithm generates a list with a number of elements equal to the number of faces present in the frame. The video module performs this procedure consecutively, so the list of faces is replaced each time a frame is analyzed.

In order to control the pointer movement, the first place in the list is used. Thus, if other people appear in the frame when the pointer control is enabled, it is possible that another face will occupy the first place in the list. This situation could generate an error since the reference point for head movements belongs to another face.

**Figure 10:**
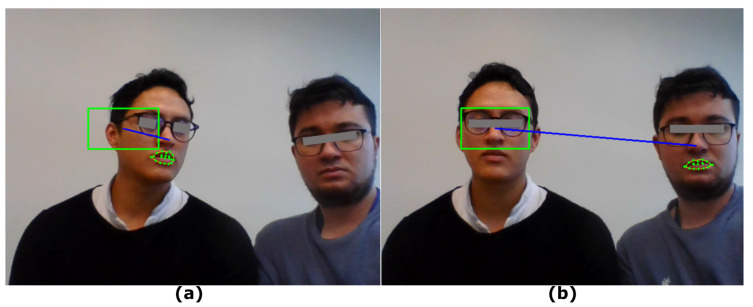
Detection with more than one face. (**a**) Image with correct operation. (**b**) Image with erroneous operation.

In Figure 10a, it can be seen that the pointer movement was correctly associated with the initial reference face. However, an erroneous operation is presented in (b), since another face different from the one which initiated the movement was detected. This will generate unwanted cursor movements, as it is preferable for a single user to be in front of the camera.

#### 5.1.3. Environmental Noise

This test was carried out while two people had a conversation near the emulator. This was done to determine the extent to which noise and other voices affect command execution. As was supposed, environmental noise affected EMKEY performance due to the sensitivity of the microphone and the noise level.

#### 5.1.4. Performance Tests in Windows OS

In this section, performance tests of the emulator in computers with different hardware and software features are presented. For this test, an executable file was generated and run on several computers with different versions of Windows OS. Table 5, presents metrics related to computational cost, cursor movement speed, response speed, use of external hardware, and correct operation of voice commands.

According to results presented in Table 5, it can be seen that EMKEY performed well in most cases whether the computers are low-, medium-, or high-end, although there are more problems in low-end devices. However, the Biostar H61MHB computer showed good results despite its features. In the case of the Lenovo 81D6 computer, during the test, it was very busy with applications running in the background. In spite of the slowness of the cursor movement and response to voice commands, the program was successfully executed without generating errors. By analyzing the devices in which the interface could not run, it was possible to determine the minimum operating parameters, which mainly depend on processor quality, OS version, and RAM. Thus, the minimum requirements are: Windows 8 OS, a processor better than or equal to an Intel Core i3 or AMD A9, and at least 6 GB of RAM. Regarding the computational cost, it can be seen that in well-maintained computers, around 20% of the CPU resources were used, which does not represent any problem when using the computer. The need to use an external webcam or microphone is related to the type of computer in which the program is executed. In low-end computers, external devices were necessary. For high-end computers, the embedded camera and microphone were enough.

#### 5.1.5. Performance Tests on Other Platforms

**Figure 11:**
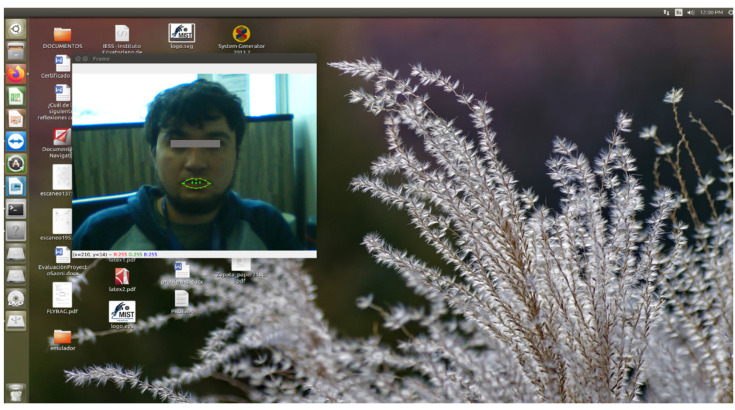
EMKEY running on Ubuntu.

To verify if EMKEY works with another operating system, a functional test was performed on Ubuntu (Figure 11). In general, most commands were executed without any issue; others needed some minor adjustments to be adapted to the visual environment of the OS. The pointer speed and the voice command response were adequate. Hence, it was possible to verify that the system works well on Ubuntu.

Furthermore, the performance of EMKEY was proved in an embedded system. Several tests were carried out on a Raspberry Pi 3B board equipped with 1 GB RAM and Raspberry Pi OS (32-bit). During the tests, the program ran slower than expected. Thus, the mouth had to be kept open for a longer time to activate the control of the pointer. The pointer speed was very slow, to the point of being uncomfortable. Concerning voice command detection, good results were obtained despite the response time. To sum up, the computational cost was high since the application occupied 52% of the computer’s capacity. Therefore, the performance of EMKEY in this board was poor due to its limited RAM capacity. Nevertheless, it is presumed that in current models, such as Raspberry Pi 4, which implements 8GB of RAM memory, the response will be better.

### 5.2. User-Testing Empirical Study

The research was made up of two independent studies in which an exploratory analysis of the usability of the emulator was carried out among participants with and without disabilities. Study 1 had a sample of 30 participants, which allowed us to identify characteristics regarding the conditions of the emulator related to the computational skills of the participants. In this study, the sample we worked with showed a large effect size of *d* = 0.80, an error probability of α=0.05, and a statistical power of 1 – β=0.75, which indicates an acceptable statistical power for the present study. On the other hand, the second study sought to work with people with disabilities, who have a very high effect size, so accessing people with these characteristics is very complicated. That is why the study’s statistical power is considered acceptable for an exploratory study of the developed emulator.

#### 5.2.1. Study 1

**Participants**: In this first study, we worked with a sample of 30 participants between the ages of 21 and 82 (*M* = 38.96, *SD* = 17.71). Regarding gender, 13 (41.9%) were women and 18 (58.1%) were men. All participants live in Quito, Ecuador, and consented to voluntary participation in the study.

**Data analysis**: The statistical analysis was performed with SPSS software based on the questions listed in Table 3. The five hypotheses proposed in the methodology section are analyzed below.

**Hypothesis 1.**  *The level of previous computer skills is associated with adaptation to the emulator.*

**Figure 12:**
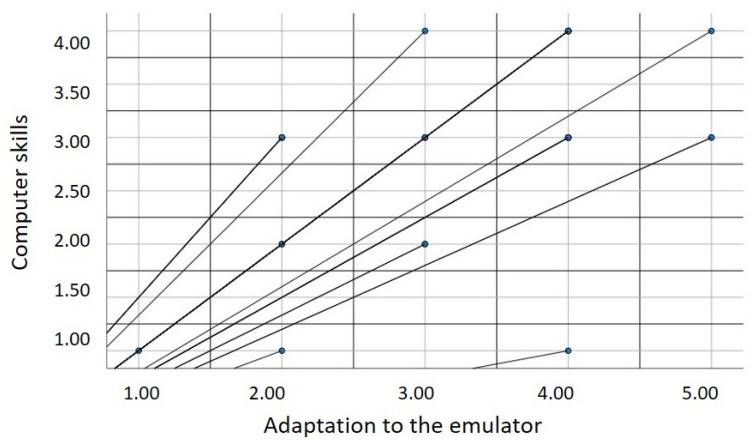
Relationship between prior computer skills and adaptability to the emulator.

The analysis performed revealed a statistically significant correlation between previously acquired computer skills and adaptation to the emulator, with a median magnitude of *r* = 0.55, *p* = 0.002 (see Figure 12). The dynamics between the variables analyzed suggest a positive correlation, indicating that as previous computer skills increase, so does the level of the user’s adaptability to the emulator.

**Hypothesis 2.**  *The time it takes to perform an internet search is associated with the age of the participants.*

**Figure 13:**
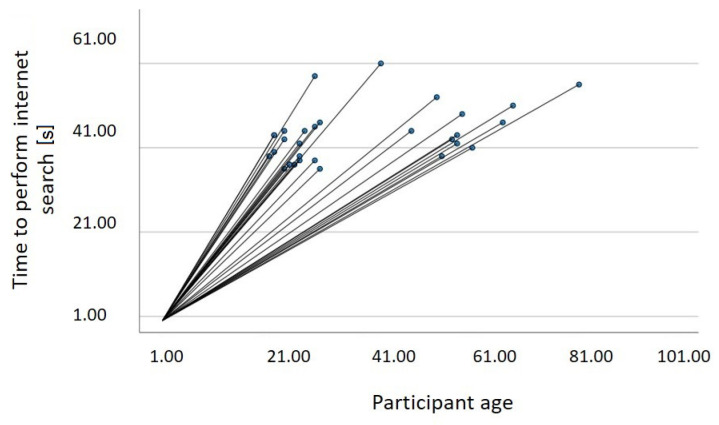
Relationship between age and the time it takes to perform an internet search.

In the statistical analysis carried out, it was found that there is a statistically significant relationship between the participants’ age and the time taken to perform the query, with *r* = 0.46, *p* = 0.01 (see Figure 13). This positive correlation means that as the age of the users increases, it take longer for them to look up information on the internet using the emulator.

**Hypothesis 3.**  *The participant’s age is related to the number of times that the pointer movement must be restarted by holding the mouth open to perform the exercise.*

**Figure 14:**
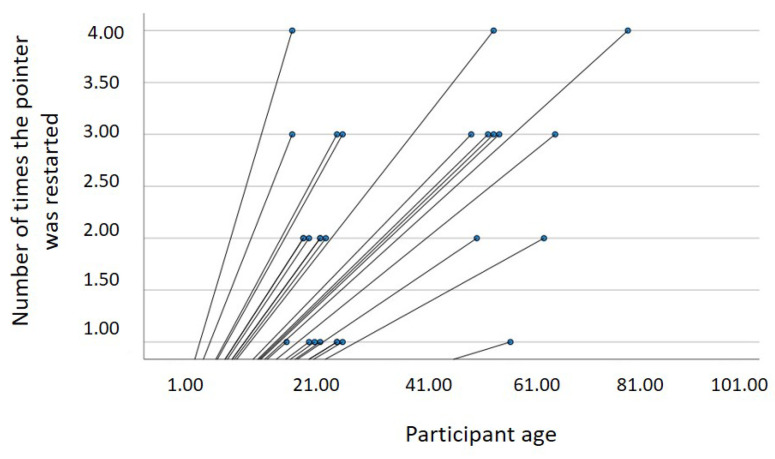
Relationship between age and number of times the pointer was restarted.

In the statistical analysis carried out, it was found that there is a statistically significant relationship between age and the number of times the pointer was restarted, with *r* = 0.40, *p* = 0.02 (see Figure 14). This positive correlation means that as the age of the users increases, the number of times the cursor movement must be restarted also increases.

**Hypothesis 4.**  *Age is associated with the degree of ease using the emulator.*

**Figure 15:**
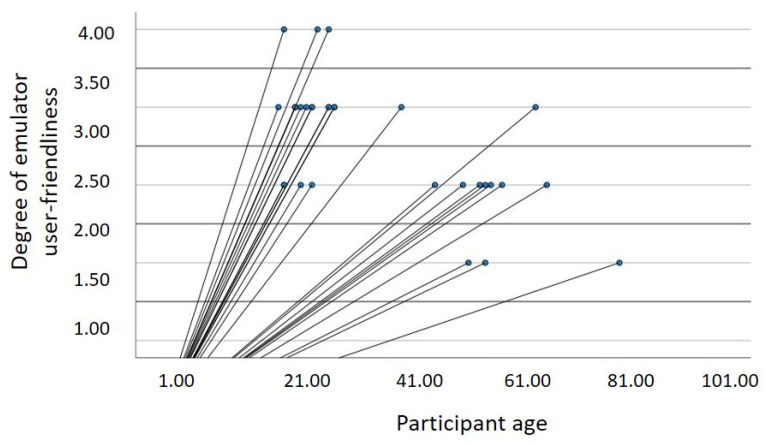
Relationship between age and ease of using the emulator.

In the analysis carried out, it was found that there is a statistically significant correlation between age and the degree of ease of using the emulator, with *r* = −0.56, *p* < 0.001 (see Figure 15). This means that the younger the users are, the easier it is for them to use the emulator. As expected, elderly people have greater difficulty.

**Hypothesis 5.**  *The performance of men and women using the emulator is similar.*

**Figure 16:**
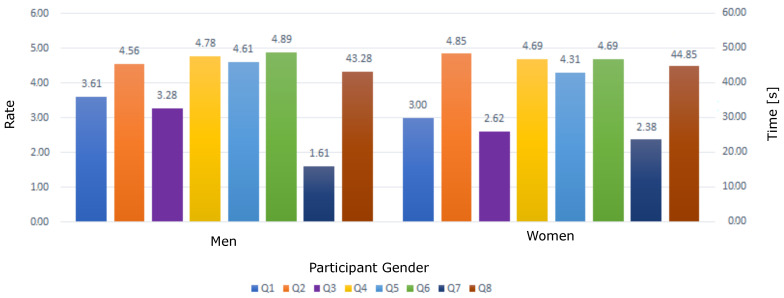
Performance of men and women using the emulator. Questions are listed in Table 3.

When comparing the answers to the survey according to the gender of the participants, it was found that there are statistically significant differences in Q1 (t (29) = 3.05, *p* = 0.002), Q3 (t (29) = 3.05, *p* = 0.001), and Q7 (t (29) = 2.99, *p* = 0.003) (see Figure 16). The questions considered for this analysis are listed in Table 3. As we can see, the results show that men have more computer skills than women; thus, adaptation to the emulator for men was easier.

#### 5.2.2. Study 2

**Participants**: The performance of the emulator was analyzed with a sample of four participants with motor disabilities: cerebral palsy, upper limb paralysis, spina bifida, and Duchenne muscular dystrophy. Regarding gender, three (75%) participants were women and one (25%) was a men. Participants’ ages ranged from 11 to 40 years old (*M* = 22.75, *SD* = 13.88). All participants had preserved cognitive status and consented to voluntary participation in the study.

**Data analysis**: Table 6 presents an assessment of the EMKEY emulator based on the answers from people with disabilities. Each question attempts to measure the usability or usefulness of the emulator. For instance, in Q2, two people think that the emulator is fair, whereas two people think that it is useful.

**Figure 17:**
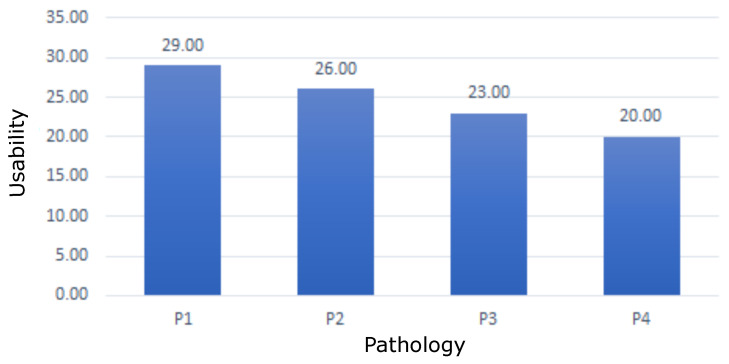
EMKEY usability according to the type of disability. P1: cerebral palsy, P2: upper limb paralysis, P3: spina bífida, P4: Duchenne muscular dystrophy.

Figure 17 indicates the mean usability by pathology. Each question in Table 6 has a maximum score of 5 points. The highest score corresponds to a positive evaluation of the question. By adding the punctuation of the questions, the emulator usability variable was constructed. It yielded results of *Min* = 20.00/35.00, *Max* = 29.00/35.00, *M* = 24.50, *SD* = 3.87. From the results, it can be seen that participants considered EMKEY to be quite useful and quite easy to use.

#### 5.2.3. Remarks Made by Participants after the Tests

When conducting the tests, it was possible to identify some aspects that should be considered for future EMKEY improvements:

- Motricity makes movement difficult since, due to the condition of the disabled user, involuntary movements are generated (participant with cerebral palsy);
- Voice commands need to be easier (participant with cerebral palsy);
- The voice tone for activating the system must be loud (participant with spina bifida).

## 6. Discussion

This section presents a comparison of the proposed emulator with other related works. Aspects such as functionality, versatility, interaction, and supported platforms are considered. Table 7 summarizes the comparison. Regarding verbal interaction, three parameters were considered: voice commands, dictation mode, and need for an internet connection. It can be observed that the majority of the referenced works implement a voice command function. Concerning the implementation of dictation mode, very few works combine it with voice commands. EMKEY implements dictation mode with the purpose of increasing the usefulness of the interface, taking into account that accessing keyboard functionalities is just as important as accessing mouse functionalities. Regarding gestural interaction, it was found that half of the cited works use a camera for detecting gestures, while the others use external devices. Considering the target application, most systems are designed for general purpose use instead of being devoted to particular applications, such as Facebook or WhatsApp.

The advantages of EMKEY lie in its simplicity and low cost, since it does not need external devices such as glasses, sensors, or electrodes, which can be obtrusive. In addition, EMKEY is intended to work on a wide range of computers to allow people to profit from their existing machines. EMKEY is not focused on a specific application; rather, its use is general, and it was tested on Windows and Linux (Ubuntu) operating systems. The interface achieves a fluid pointer movement thanks to screen segmentation via voice commands, without the need for an internet connection. The implementation of the dictation mode allows the user to replace the use of a keyboard for simple tasks. In addition, EMKEY can interact with free open-source tools such as *OptiKey*, which is an on-screen keyboard, to complement the dictation mode.

Regarding EMKEY usefulness, a test was conducted to compare EMKEY’s performance with that of a common mouse and keyboard. The results show that it takes more than three times longer to execute the same internet search with EMKEY (44 s) than with standard peripherals (12 s). It is clear that the aim of this proposal is not to replace the use of the mouse and keyboard, but rather to provide people with motor disabilities a chance to improve their interaction with the computer. Hence, it is expected that EMKEY will allow them to perform tasks such as opening files, surfing the internet, making inquiries, watching videos, checking emails, or using social media. However, the interface is not suitable for applications requiring constant and continuous use of the mouse, such as video games.

The main limitation of EMKEY is related to its speech-to-text functionality since it has been implemented through standard dictation/transcription libraries. Errors must then be corrected by the user. Consequently, the use of this mode is not efficient enough for writing formal text. The current version of EMKEY uses Spanish language libraries, which allow dictation/transcription in Spanish. However, if another language is required, it is as simple as configuring the libraries in the desired language (if available). Lastly, there are some minimal features that must be supported to guarantee adequate operation. For Windows OS based computers, the minimum requirements are an Intel Core I3 processor or similar, 6GB of RAM memory, and Windows version 8.

## 7. Conclusions

This work describes the design, implementation, and validation of a mouse and keyboard emulator called EMKEY, devoted to users with limited mobility in the upper limbs. The emulator is based on artificial vision and voice recognition. Voice commands were used not only for performing mouse and keyboard actions, but also to speed up the pointer movement by means of screen segmentation. Regarding the emulator’s performance, evaluations under different lighting conditions showed that EMKEY can work in dimly lit environments as long as the user’s face is well lit. EMKEY functionalities were tested in computers with different hardware and operating system features, which ensures its wide usability. Concerning computer peripherals, no external camera is needed to achieve good face detection, except with low-end computers. However, an external microphone is suggested to improve voice detection.

EMKEY evaluation was carried out considering two groups of users: a group of 30 participants without physical disabilities and a group of four participants with upper limb disabilities. Unfortunately, for the second group, it was very difficult to include more participants since special permission was required to have them take part in the testing. However, it should be considered that the size effect of the motor paralysis variable is large. Therefore, working with a small sample size is acceptable in this type of exploratory work on technological development to support disabled people. In addition, the statistical potential for a first study with the developed emulator was acceptable, and the results provide valuable insights for future studies on this technological innovation.

Concerning the results, it can be statistically shown that the ease of use and adaptation regarding EMKEY are proportional to age, as well as previous computer knowledge. For people with reduced mobility in the upper limbs with no computer experience, the adaptation process took longer and required more learning sessions. Nevertheless, once having moved beyond this adaptation stage, the participants considered EMKEY to be a handy tool that gives them the opportunity to access to different resources through the computer. In addition, these studies suggest that the emulator’s performance is affected by the participant’s gender. For instance, the results show that women have fewer computing skills; thus, they require more adaptation time than men.

Finally, the main contribution of this work was to develop a low-cost, fairly accurate, and versatile mouse emulator compatible with a wide range of computers that includes dictation and transcription functions with no need for an internet connection.

In future work, the EMKEY emulator will be improved by taking into account the results obtained from previous and new tests. In addition, EMKEY will be combined with other free open-source tools to extend its functionality. As the interaction of EMKEY with *OptiKey* yielded promising results, it is planned to integrate and test the interaction with some software package such as *Dasher* or *Project Iris* to allow the computer to be entirely controlled with the eyes. Furthermore, EMKEY will be tested in other single-board computers (SBCs) such as Raspberry Pi 4, Odroid XU4, and, Libre board AML-S905X-CC. 

## Figures and Tables

**Table 1:** Predefined dictation commands.

Command	Function
“borrar” (delete)	Delete 1 character.
“borrar dos” (delete two)	Delete 2 characters.
“borrar tres” (delete three)	Delete 3 characters.
“borrar cuatro” (delete four)	Delete 4 characters.
“borrar cinco” (delete five)	Delete 5 characters.
“borrar diez” (delete ten)	Delete 10 characters.
“borrar mucho” (delete a lot)	Delete 40 characters.
“espacio” (space)	Generate a space.

Note: Command translation in parentheses.

**Table 2:** EMKEY predefined control commands.

Command	Function
“uno” (one)	Move the cursor to the first quadrant.
“dos” (two)	Move the cursor to the second quadrant.
“tres” (three)	Move the cursor to the third quadrant.
“cuatro” (four)	Move the cursor to the fourth quadrant.
“cinco” (five)	Move the cursor to the fifth quadrant.
“seis” (six)	Move the cursor to the sixth quadrant.
“siete” (seven)	Move the cursor to the seventh quadrant.
“ocho” (eight)	Move the cursor to the eighth quadrant.
“nueve” (nine)	Move the cursor to the ninth quadrant.
“diez” (ten)	Move the cursor to the tenth quadrant.
“once” (eleven)	Move the cursor to the eleventh quadrant.
“doce” (twelve)	Move the cursor to the twelfth quadrant.
“inicio” (beginning)	Click on the start icon in the left corner.
“escritorio” (desktop)	Click in the lower right corner on the quick access bar of the desktop in Windows and position the cursor in the center of the screen (exclusive for Windows).
“centro” (center)	Position the cursor in the center of the screen.
“clic” (click)	Click.
“doble” (double)	Double-click.
“derecho” (right)	Right-click.
“sujetar” (hold)	Hold left click.
“soltar” (release)	Release left click.
“cerrar” (close)	Close the current window.
“minimizar” (minimize)	Minimize the current window.
“ventanas” (windows)	Open the menu of open windows (exclusive for Windows).
“enter”	Perform the function of the “ENTER” key.
“dictado” (dictation)	Activate dictation mode.
“salir dictado” (exit dictation)	Deactivate dictation mode.
“apagar” (turn off)	Close the program.

Note: Command translation in parentheses.

**Table 3:** List of survey questions.

Question	Description
Q1	How advanced are your computer skills?
Q2	How functional do you think the emulator is for people with upper limb disability?
Q3	How easy was the adaptation to the use of the emulator?
Q4	How accurate do you think the detection of voice commands is on your computer?
Q5	How accurate do you think the detection of your face was?
Q6	How useful have you found the screen segmentation commands?
Q7	How many times did you have to restart the cursor movement?
Q8	Using a stopwatch, measure the time it takes to make the query “world’s most populated country” in Google and open the first link that appears in the search engine. The time is measured in seconds.

**Table 4:** List of modified survey questions for people with motor disability.

Question	Description
Q7b	Do you think that the cursor movement speed is adequate to select small objects?
Q8b	How easy was the exercise?

**Table 5:** Test results using computers with different features.

Computer	Processor	RAM	Operating System	Computational Cost	Expected Cursor Speed	Voice Command Response	All Commands Works	External Webcam	External Microphone	Problems
Biostar H61MHB	Intel Core i3-3220	6GB	Windows 8	23%	Yes	Very good	Yes	Yes	Yes	None
Lenovo 81D6	AMD A9-9425	8GB	Windows 10	47.80%	No	Fair	Yes	No	Yes	Saturated computer
HP–HP Laptop 15-db1xxx	AMD Ryzen 3 3200U	8GB	Windows 10	24%	Yes	Good	Yes	No	No	None
Toshiba Satellite L 15W-B	Intel Pentium N3540	4GB	Windows 10	-	-	-	-	-	-	Program could not be executed
Dell Inspirion 15 3515	AMD Ryzen 5 3450U	8GB	Windows 11	24.20%	Yes	Very good	No	No	Yes	None
ASUS Vivobook 15	Intel Core i5-8250	8GB	Windows 10	22%	Yes	Very good	Yes	No	Yes	None
Dell G3 3579	Intel Core i7-8750H	16GB	Windows 10	15.40%	Yes	Very good	Yes	No	No	None
Toshiba Satellite P55W-C	Intel Core									
i7-6500U	12GB	Windows 10	32.30%	Yes	Very good	Yes	No	No	None	
Toshiba Satellite P75-A7200	Intel Core i7-4700	8GB	Windows 8	13.8%	Yes	Very good	Yes	No	No	None
ASUS K53E	Intel Core i7-2670	8GB	Windows 7	-	-	-	-	-	-	Program could not be executed

**Table 6:** Answer distribution in agreement with the four participants’ responses in Study 2.

Question	Assessments		
How good are your computer skills?	Poor 1 (25%)	Fair 1 (25%)	Very good 2 (50%)
How useful do you think the emulator is for people with upper limb disability	Somewhat useful 2 (50%)	Useful 2 (50%)	
How easy was the adaptation to the use of the emulator?	Somewhat easy 1 (25%)	Easy 2 (50%)	Very easy 1 (25%)
How accurate do you think the detection of voice commands is?		Good 1 (25%)	Very good 3 (75%)
How accurate do you think the detection of your face was?		Good 2 (50%)	Very good 2 (50%)
How useful have you found the screen segmentation commands?			Very useful 4 (100%)
Do you think the cursor movement speed is adequate to select small objects?	No 1 (25%)	Yes 3 (75%)	
How easy was the exercise?	Somewhat easy 3 (75%)	Easy 1 (25%)	

**Table 7:** Comparison table of EMKEY with other works.

Referencia	Interaction					Versatility		Supported Devices	
	Verbal			Gestural		Works on Specific Applications	General Purpose Applications	Computer	Mobile
	Voice Commands	Dictation	Need Connection	Webcam	Others				
This work (EMKEY)	x	x	No	x	-	-	x	x	-
Lupu et al. [4]	-	-	-	-	x	-	x	x	-
Zhang et al. [5]		-	-	-	x	-	x	x	-
Sias et al. [6]	-	-	-	-	x	-	x	x	-
Sinha et al. [17]	x	-	Yes	x	-	-	x	x	-
Loewenich & Maire [16]	x	x	Yes	x	-	-	x	x	-
Mosquera et al. [8]	x	-	Yes	x	-	x	-	x	-
Mosquera et al. [9]	x	x	Yes	x	-	x	-	x	-
Ferrin et al. [10]	x	-	Yes	x	-	x	-	x	-
Khan et al. [19]	-	-	-	x	-	-	x	x	-
Guiawal et al. [18]	-	-	-	x	-	-	x	x	-
Gupta et al. [20]	x	-	No	x	-	-	x	x	-
Darabkh et al. [22]	x	-	No	-	-	-	x	x	-
Paudyal et al. [24]	x	-	Yes	-	x	x	-	x	-
Lund et al. [11]	-	-	-	-	x	-	x	x	-
Sahadat and Ghovanloo [12]	-	x	Yes	-	x	-	x	x	-
Sumak et al. [15]	-	-	-	-	x	-	x	x	-
Wang et al. [25]	x	-	Yes	-	-	x	-	-	x
Lancioni et al. [26]	-	-	-	-	x	x	-	-	x

## Data Availability

The data used to support the findings of this study are available from the corresponding author upon request.

## Abbreviations

The following abbreviations are used in this manuscript:

EAR	Eye aspect ratio
EEC	Electroencephalography
EMG	Electromyography
EMKEY	Emulator of mouse and keyboard
MAR	Mouth aspect ratio
MP3	MPEG-1 Audio Layer III
OS	Operating system
RAM	Random access memory
SBC	Single-board computer
SD	Standard deviation
